# New Stereoacuity Test Using a 3-Dimensional Display System in Children

**DOI:** 10.1371/journal.pone.0116626

**Published:** 2015-02-18

**Authors:** Sang Beom Han, Hee Kyung Yang, Jonghyun Kim, Keehoon Hong, Byoungho Lee, Jeong-Min Hwang

**Affiliations:** 1 Department of Ophthalmology, Kangwon National University Hospital, Kangwon National University Graduate School of Medicine, Chuncheon, Korea; 2 Department of Ophthalmology, Seoul National University Bundang Hospital, Seoul National University College of Medicine, Seoul, Korea; 3 School of Electrical Engineering, Seoul National University, Seoul, Korea; UMR8194, FRANCE

## Abstract

The previously developed 3-dimensional (3D) display stereoacuity tests were validated only at distance. We developed a new stereoacuity test using a 3D display that works both at near and distance and evaluated its validity in children with and without strabismus. Sixty children (age range, 6 to 18 years) with variable ranges of stereoacuity were included. Side-by-side randot images of 4 different simple objects (star, circle, rectangle, and triangle) with a wide range of crossed horizontal disparities (3000 to 20 arcsec) were randomly displayed on a 3D monitor with MATLAB (Matworks, Inc., Natick, MA, USA) and were presented to subjects wearing shutter glasses at 0.5 m and 3 m. The 3D image was located in front of (conventional) or behind (proposed) the background image on the 3D monitor. The results with the new 3D stereotest (conventional and proposed) were compared with those of the near and distance Randot stereotests. At near, the Bland-Altman plots of the conventional and proposed 3D stereotest did not show significant difference, both of which were poorer than the Randot test. At distance, the results of the proposed 3D stereotest were similar to the Randot test, but the conventional 3D stereotest results were better than those of the other two tests. The results of the proposed 3D stereotest and Randot stereotest were identical in 83.3% at near and 88.3% at distance. More than 95% of subjects showed concordance within 2 grades between the 2 tests at both near and distance. In conclusion, the newly proposed 3D stereotest shows good concordance with the Randot stereotests in children with and without strabismus.

## Introduction

Stereoacuity is defined as the smallest amount of horizontal retinal disparity that provokes perception of depth or stereopsis.[1,2] Diseases associated with disruption of normal binocular fusion, such as amblyopia, strabismus, anisometropia or aniseikonia, lead to decreased stereoacuity.[3] Stereoacuity tests could be used for the evaluation of those diseases. The most commonly used stereoacuity tests are polaroid vectograph tests, in which the two eyes are dissociated and each eye is presented with a separate view of 2-dimensional objects that are shown to each eye, such as, “random dot” tests.[4] However, the currently available polaroid vectograph stereoacuity tests have drawbacks as follows: 1) Stereoacuity worse than 400–800 seconds of arc (arcsec) cannot be measured,[5,6] 2) The small numbers of predefined intervals of these stereotests are not standardized and might limit the precision of the measurement, which uses different levels of disparities at near and distance that cannot be compared directly,[7] 3) Learning effect of fixed answers can decrease the reliability of repetitive stereotests,[7] 4) Only black and white targets with high contrast are applied, 5) Contour-based tests are with high risk of monocular cues.[8]

In our prior study, we developed a new stereotest using a 3-dimensional (3D) display, and showed its efficacy in normal adult subjects, which showed good concordance with the Distance Randot stereotest (version 2; Stereo Optical Co, Inc. Chicago, IL, USA) and relatively good test—retest reliability of less than 5% of subjects showing a 2-grade or more difference between the 2 tests.[7] However, the study had several limitations as follows: First, it was limited to young adult subjects and tested only at a distance of 3 m. As stereoacuity thresholds can be affected by retinal eccentricity,[9] circle size and distance between circles should be adjusted proportionally according to the test distance. Second, near stereoacuity tests have a potential mismatch of accommodation and convergence in the stereoscopic display,[10,11] thus new methodologies should be adopted for the development of a reliable stereoacuity test to be performed both at near and at distance. Third, the prototype test failed to completely exclude monocular cues especially having a high contrast contour-based circle stereogram. Lastly, the subjects had to press the corresponding position of the stimulus on a keypad which is difficult for young children to perform.

To overcome the limitations of our previous study, we developed a new 3D stereotest using a 3D monitor and proposed a novel method of stereoscopic display. The size and distance between stereograms were adjusted according to the measurement distance and the contours of stereograms were eliminated to exclude monocular cues at larger disparities. Answering methods were also simplified to make it easy for children to perform the test. We evaluated the efficacy of the proposed 3D stereotest compared to the conventional 3D stereotest and Randot stereoacuity test both at near and distance in children with and without strabismus.

## Methods

### Ethics Statement

This study was conducted in compliance with the Declarations of Helsinki and was approved by the Institutional Review Board of Seoul National University Bundang Hospital.

### Participants

Sixty children (age range, 6 to 18 years) who visited the pediatric ophthalmology and strabismus department of Seoul National University Bundang Hospital from March, 2012 to February, 2013 were included. The inclusion criteria were as follows: (1) Best-corrected visual acuity of 20/25 or better in both eyes (2) Subjects with orthotropia or eso-/or exotropia by alternate prism cover tests (3) No amblyopia and (4) No history of intraocular surgery or intraocular pathologies that can affect vision, such as, cataract, glaucoma, or retinal abnormalities. All subjects underwent evaluation including best-corrected visual acuity with the Snellen Chart, cycloplegic refraction, alternate prism cover test with fixation targets at 0.33 and 6 m, Randot stereotest (Stereo Optical Co, Inc. Chicago, IL, USA) at 0.4 m, Distance Randot stereotest (Stereo Optical Co, Inc. Chicago, IL, USA) at 3 m, and the new 3D stereotest at 0.5 m and 3 m.

### Standard Randot stereotests

Randot stereotest (Stereo Optical Co, Inc. Chicago, IL, USA) was performed at near and the Distance Randot stereotest (Stereo Optical Co, Inc. Chicago, IL, USA) at distance (3 m). The visual stimulus of the Randot stereotest is contour-based circles and animals. The visual stimulus of the Distance Randot stereotest is random dot-based geometrical shapes without contours including star, circle, rectangle, and triangle. The dot size of the Distance Randot stereotest is approximately 2 mm, which is similar to the pixel dot size of the 3D stereotests of 0.199mm. The image size of the stereogram circle is 8.5 mm in the Randot stereotest circle and 90 mm in the Distance Randot stereotest. The largest disparity level was tested and, if the patient was correct, moved on to a 1 level smaller disparity. The smallest disparity at which a subject identified shapes correctly was recorded as the stereoacuity threshold.

### Randot stereoscopic images of the 3D stereotests

**Fig 1:**
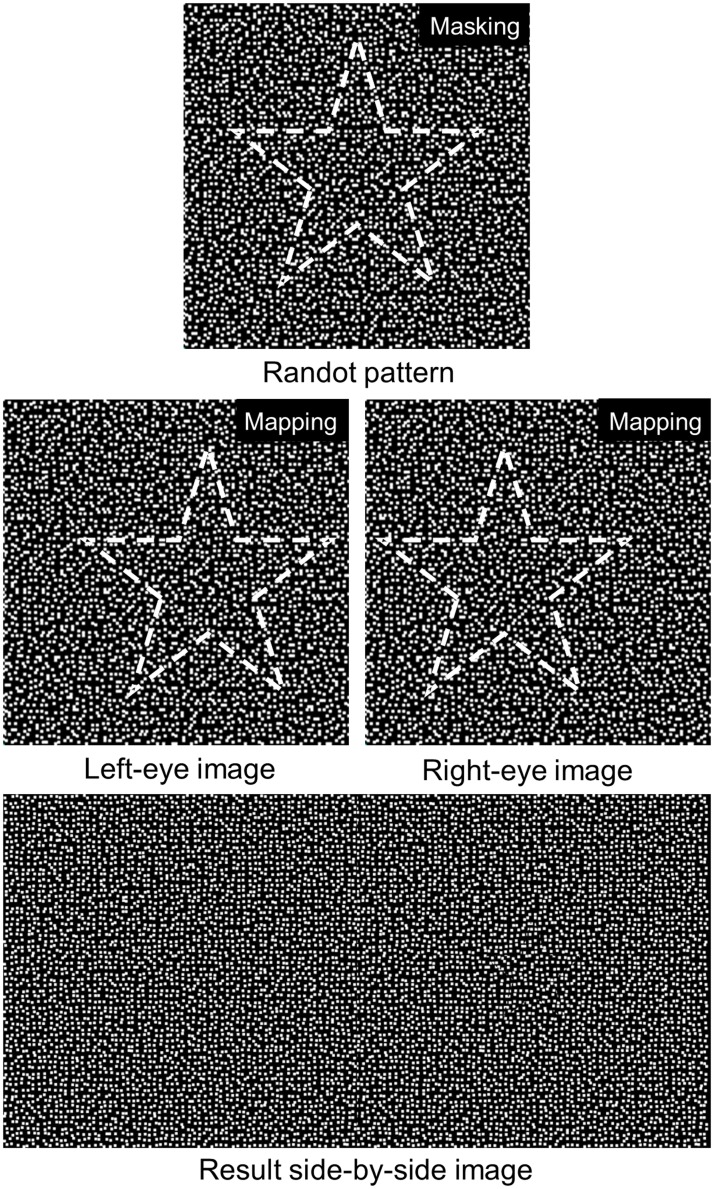
An example of generating a side-by-side Randot stereogram image with a ‘star’.

Randot stereogram images with a wide range of crossed horizontal disparities (3000 to 20 arcsec) were displayed on the 3D monitor (17 inch Full-HD 3D; Dell, Round Rock, TX, USA). Side-by-side randot images of 4 different simple objects (star, circle, rectangle, and triangle) were pre-generated with MATLAB (Matworks, Inc., Natick, MA, USA). An example of the side-by-side image generation is shown in Fig. 1. A uniform randot pattern was generated with random function. The size of the dot is 0.199mm, identical to the pixel size, and the dots are distributed in every two pixels. Therefore, a dot is located randomly in every 2 by 2 pixel square. Then, the object mask was covered, and the mask split inside and outside randot patterns shown in Fig. 1. According to the target horizontal disparity, the inside and outside randot patterns were properly located in the left-eye and right-eye image. Since pixels are quantized and the pixel pitch is not small enough to provide small disparities, the sub-pixel rendering method was applied. By shifting the randot patterns in sub-pixel scale, the effective pixel pitch can be reduced to 1/3. These images formed a side-by-side image as shown in Fig. 1. For example, to express the smallest disparity, we drew a pixel with the R, G, and B subpixel in one pixel for the left-eye view, and with G and B subpixel in a pixel and R subpixel in the next pixel for the right-eye view. Since our images consist of only a number of black and white dots, the subpixel-shift technique does not cause additional image degradation problems. The result image without a contour clearly eliminated the monocular cues with uniformly distributed randots, and randot stereogram images with different randot sizes were generated.

### Shutter glass type stereoscopic 3D display

The stimulus was presented on a 17-inch shutter glass type stereoscopic monitor (17 inch Full-HD 3D; Dell, Round Rock, TX, USA) with 355 cd/m^2^, resolution of 1920 × 1080 pixels, and contrast ratio of 847:1. We used LCD type shutter glasses (3D vision kit, NVIDIA) to separate the stereoscopic images. The right-eye and left-eye images were shown on the display sequentially with a frame rate of 120 Hz. The subject wore synchronized shutter glasses, thus the glass in front of each eye was opened alternately when the image corresponding to the eye was shown on the display panel. The display worked with a side-by-side stereoscopic image. However, a shutter glass type 3D display suffers from cross-talk, and the cross-talk would impair the depth perception.[12] This unintended errors can be reduced with a backlight control method,[13] or a glasses-free cross-talk reduced 3D display system.[14]

### 3D stereoacuity test

Four kinds of randot images with crossed disparity (3000 to 20 arcsec; 16 different disparity levels as follows: 3000, 2000, 1000, 800, 600, 400, 250, 200, 140, 100, 70, 50, 40, 30, 25 and 20arcsec; MATLAB; Matworks, Inc., Natick, MA, USA) were generated on the 3D monitor using shutter glasses and were presented to subjects at 0.5 and 3 m. However, at the testing distance of 0.5 m, due to the quantized pixel and the pixel pitch, the smallest angular disparity provided by our system is approximately 30 arcsec. Therefore the test in near distance provides 12 different disparity levels from 3000 to 30 arcsec: 3000, 2000, 1000, 800, 600, 400, 250, 200, 140, 100, 70 and 30.

**Fig 2:**
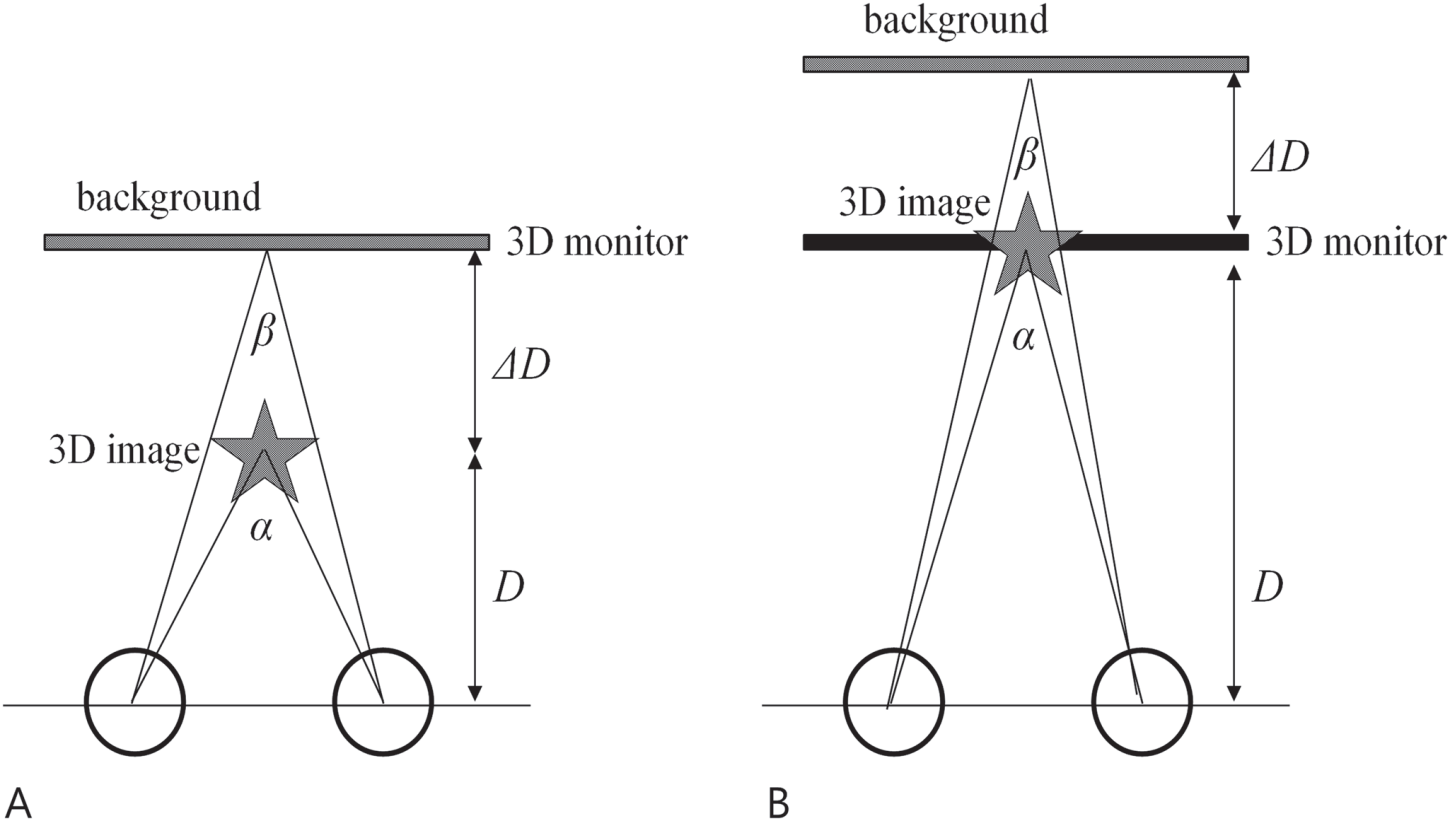
New stereotest using a 3-dimensional (3D) display with the (A) conventional method and (B) proposed method. (A) The conventional stereotest using a 3D monitor display. The 3D monitor and shutter glasses cause binocular disparity and the subject feels the difference in depth between the stereoimage and the background as shown. In this case, the stereoimage is located in front of the 3D monitor panel and the background image is located at the 3D monitor. (B) The proposed stereotest using a 3D monitor display. The 3D image is located at the 3D monitor panel and the background image is located behind the 3D monitor as shown.

At each distance, two different types of stereotests were performed: the conventional method and the proposed method. Briefly, in the conventional method, 3D images were located in front of the 3D monitor panel and the background image was located on the 3D monitor. By contrast, in our proposed method, the 3D image was located at the level of the 3D monitor panel and the background image was located behind the 3D monitor (Fig. 2). While the image of the stimulus changed randomly among 4 possible images, the subjects were instructed to answer which image they perceived stereoscopically to the instructor and the instructor pressed the correct or incorrect button, according to the answer. If the subject identified the shape correctly at the first level of disparity (3000 arcsec) at distance, half of the remnant levels (7 out of 15 disparity levels) were skipped and went directly to the next disparity level of 140 arcsec. If the test subject was wrong, the subject was tested with a 1 level larger disparity (200 arcsec), consecutively, and so on. The smallest disparity at which a subject identified shapes correctly was recorded as the stereoacuity threshold. The test results were compared with those from Randot stereotests at both near and distance, respectively.

The test-retest variability for most stereoacuity tests is known to be more than 2 octaves (quadrupling of threshold arcsec).[15] Changes of less than 2 octaves cannot be distinguished from test-retest variability, and caution should be taken in interpreting the results. Therefore, we grouped stereoacuity levels into categories larger than the test-retest variability of the Randot stereoacuity test, especially for categories of subnormal stereoacuity.[15] Stereoacuity values were grouped into 3 categories as normal (20 to 200 arcsec), coarse (250 to 400 arcsec), and poor (> 400 arcsec), according to the literature.[4]

### Statistical analyses

Continuous values were expressed as mean ± standard deviation. Stereoacuity results were transformed to log arcsec for analyses. Pearson’s correlation coefficients were evaluated to determine the validity of the new 3D stereotest compared to the Randot stereotests. To evaluate the concordance of the 3D stereotest and the Randot stereotest at both near and distance, stereoacuity values were grouped into 3 categories as normal (20 to 200 arcsec), coarse (250 to 400 arcsec), and poor (> 400 arcsec). The proportion of subjects with a categorical stereoacuity difference of more than 1 or 2 levels between the proposed 3D stereotest and Randot stereoacuity test at near and distance were evaluated. Agreement between scores also was represented as Bland—Altman plots. Differences between test and retest scores were calculated for each subject using the results of the Randot stereotest and proposed 3D stereotest at near and distance. The 95% limits of agreement were calculated. These values then were converted back to octave steps, which also can be described as doublings.[15] Each doubling of the stereoacuity threshold corresponds to a change of 0.3 in the log transformed value; therefore, we divided the 95% limit of agreement values by 0.3 to calculate the number of octaves.[7,15] Results were interpreted as statistically significant when *P* values were less than 0.05.

## Results

### Participants

The mean age of subjects was 11.3±2.5 years (range, 6–18 years of age). Twenty patients with orthotropia, 14 esotropes with a mean deviation of 13.0±7.1 prism diopters (PD) (range, 6~30 PD) and 26 intermittent exotropes with a mean deviation of 12.3±5.8 PD (range, 4~30 PD) were included in the study. The mean spherical equivalent refractive error was-1.81±3.27 D (range, -9.50~ +4.63 D).

### Concordance between stereoacuity tests

At near, stereoacuity threshold scores were 109±146 arcsec (range, 800 to 20 arcsec) using the Randot stereotest, 171±100 arcsec (range, 600 to 30 arcsec) using the conventional 3D stereotest and 148±100 arcsec (range, 600 to 30 arcsec) using the proposed 3D stereotest. At distance, stereoacuity threshold scores were 298 ± 276 arcsec (range, 800 to 60 arcsec) using the Distance Randot stereotest, 139 ± 95 arcsec (range, 600 to 25 arcsec) using the conventional 3D stereotest and 328 ± 305 arcsec (range, 800 to 25 arcsec) using the proposed 3D stereotest. A multivariate logistic regression analysis revealed that age, visual acuity, and eye alignment were not significantly correlated with the stereoacuity scores.

At near, the Randot stereotest showed a positive correlation with the proposed 3D stereotest (r = 0.424, P = 0.002), but not with the conventional 3D stereotest (r = 0.181, P = 0.166). The conventional and proposed 3D stereotests showed a positive correlation (r = 0.595, P<0.001). At distance, the Distance Randot stereotest showed a significant correlation with the proposed 3D stereotest (r = 0.446, P<0.001), but not with the conventional 3D stereotest (r = 0.191, P = 0.174). The conventional and proposed 3D stereotests showed a positive correlation (r = 0.292, P = 0.036).

**Fig 3:**
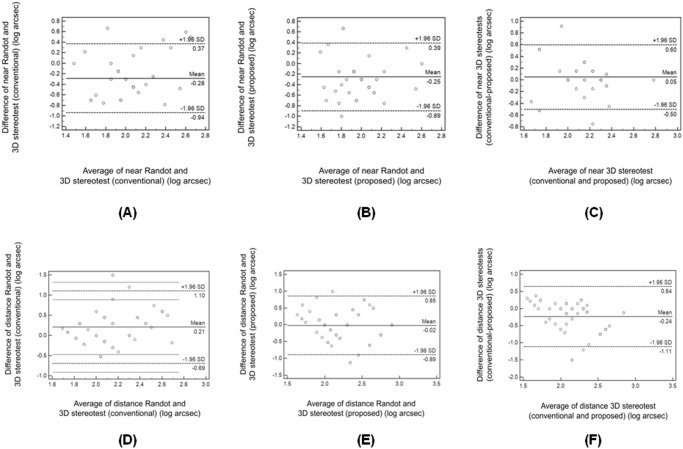
Bland—Altman plots showing the concordance between near and distance 3-dimensional (3-D) stereotests and the Randot stereotest in children. The magnitude of the test differences did not seem to be dependent on the level of stereoacuity. The concordance between the Randot stereotest and the two 3D stereotests are presented on the Bland—Altman plots in Fig. 3(A-F). The results of the conventional 3D stereotest were poorer than that of the corresponding Randot test for the near tests (mean difference: 0.28 log arcsec [0.93 octave], Fig. 3A) and better for the distance tests (0.21 log arcsec [0.70 octave], Fig. 3D). The results of the proposed 3D stereotest were poorer than that of the corresponding Randot test for the near tests (0.25 log arcsec [0.83 octave], Fig. 3B) but similar for distance tests (0.02 log arcsec [0.07 octave], Fig. 3E). The results of the conventional 3D stereotest were similar with the corresponding proposed 3D stereotest for the near tests (mean difference: 0.05 log arcsec [0.17 octave], Fig. 3C), but better for the distance tests (0.24 log arcsec [0.80 octave], Fig. 3F).

The concordance between the Randot stereotest and the two 3D stereotests are presented on the Bland—Altman plots in Fig. 3 (A-F). The results of the conventional 3D stereotest were poorer than that of the corresponding Randot test for the near tests (mean difference: 0.28 log arcsec [0.93 octave], Fig. 3A) and better for the distance tests (0.21 log arcsec [0.70 octave], Fig. 3D). The results of the proposed 3D stereotest were poorer than that of the corresponding Randot test for the near tests (0.25 log arcsec [0.83 octave], Fig. 3B) but similar for distance tests (0.02 log arcsec [0.07 octave], Fig. 3E). The results of the conventional 3D stereotest were similar with the corresponding proposed 3D stereotest for the near tests (mean difference: 0.05 log arcsec [0.17 octave], Fig. 3C), but better for the distance tests (0.24 log arcsec [0.80 octave], Fig. 3F).

Tables 1–3 show the concordance of the proposed 3D stereotest and the Randot stereotest at near and distance in children. In a categorical definition of stereopsis (normal, coarse, and poor), the results for the 2 stereotests were identical in 83.3% (50/60) at near and 88.3% (53/60) at distance. No more than 5% of subjects showed a 2-grade or more difference between the 2 tests at both near and distance. The stereoacuity data obtained from the 3D stereotest exhibited good concordance with the data from the Randot stereotest, supporting the validity of the new 3D stereotest.

**Table 1:** Classification of agreement between the Near 3D stereotest (proposed) and the Randot Stereotest by individual subjects.

Randot Stereotest (arcsec)	Near 3D stereotest (arcsec)		
	Poor (600-nil)	Coarse (250–400)	Normal (30–200)
Poor (nil)	0	1	1
Coarse (400)	0	0	2
Normal (20–200)	2	4	50^a^

The number of subjects represented by each category is embedded in each category.

^a^ Identical results on Near 3D stereotest and Randot Stereotest.

**Table 2:** Categorical agreement between the Distance 3D stereotest (proposed) and the Distance Randot stereotest by individual subjects.

Distant Randot (arcsec)	Distance 3D stereotest (arcsec)		
	Poor (600-nil)	Coarse (250–400)	Normal (20–200)
Poor (nil)	0	0	0
Coarse (400)	0	0	3
Normal (60–200)	1	3	53^a^

The number of subjects represented by each category is embedded in each category.

^a^ Identical results on Distance 3D stereotest and Distance Randot stereotest.

**Table 3:** Proportion of subjects with a categorical stereoacuity difference of more than 1 level between the proposed 3D stereotests and Randot stereotests at near and distance.

Categorical Difference ≥ 1 level	
Randot Stereotest—Near 3D stereo (proposed)	Distance Randot—Distance 3D stereo (proposed)
16.7% (10/60)	11.7% (7/60)
Categorical Difference ≥ 2 level	
Randot Stereotest—Near 3D stereo (proposed)	Distance Randot—Distance 3D stereo (proposed)
5.0% (3/60)	1.7% (1/60)

The categorical definition of stereopsis was divided into 3 steps; normal, coarse and poor.

## Discussion

In the present study, we did the following measures to develop a new stereoacuity that addresses the aforementioned problems with improved efficacy and validity. First, we used 4 types of stereo images including stars, triangles, rectangles and circles instead of Landolt rings, which were randomly presented for the comprehension of children. Second, we adjusted the density of the random dots to be uniform throughout the whole field of the images to exclude monocular cues. Moreover, for the exclusion of monocular cues, we used an active-type shutter glass 3D monitor rather than a passive Film-type Patterned Retarder polaroid 3D monitor used in our previous study.[7] An active type display utilizes a time-multiplexed technique, thus, does not have the problem found in polaroid displays of different heights according to the varying lines. Third, we performed the near stereotest at 0.5 m, and showed its efficacy and validity. As stereoacuity thresholds can be affected by retinal eccentricity,[9] circle size and distance between circles were adjusted proportionally according to the test distance. Finally, we introduced a proposed method theoretically expected to be able to reduce fixation disparity induced by convergence and accommodation. [16]

Although several researchers introduced 3D display-based stereoacuity tests, the studies had small study population, did not perform the distance stereotest, and lacked validity and reliability of the stereotests.[17–20] Recently, we introduced a distance stereotest using 3D display, and reported its validity and reliability in normal subjects.[7] In our previous study, we used a contour-based circle constructed by random dots and subjects were asked to choose one of the four positions that was perceived stereoscopically. However, this is often difficult for children to understand and respond. Thus, our prior study was conducted only for adults.[7] We also failed to rigorously exclude monocular cues, conceivably because of contour-based circles and differences in the densities of the random dots inside and outside of the Landolt rings. Moreover, the test was conducted only at 3 m and the efficacy of the 3D stereotest in measuring near stereoacuity was not evaluated.[10]

On clinical grounds, our newly proposed 3D stereoacuity test roughly showed good concordance with the Randot stereotests both at near and at distance. However, Bland-Altman plots showed that during distance viewing, the conventional 3D stereotest results were much better than both the proposed 3D stereotest and Distance Randot stereotest. Although the reason is unclear, this may be partly explained by the closer position and better visibility of the 3D image in the conventional method at long distances, which could overestimate the true stereoacuity compared to the proposed method.[21,22]

Near stereotest results were similar between the conventional and proposed methods, which failed to prove the assumption of a potential mismatch of accommodation and convergence in the stereoscopic display. The reason why near 3D stereotest results were poorer than the Randot stereoacuity test can be explained by the difference of the stereoimages used in these tests—random dot-based geometrical shapes in the 3D stereotests and contour-based circles in the Randot stereotests, respectively. The contour-based stereograms used in Randot stereotest have potential monocular cues. However, random dot-based geometrical shapes used in the 3D stereotests were generated without contours, and this may have affected the results. This phenomenon was also observed in our previous study, as the 3D stereotest results at distance using contour-based stereograms were better than the Distance Randot stereotest results using random dot-based geometrical shapes. In contrast, we generated random dot-based geometrical shapes without contours in this study, similar to the images used in the Distance Randot stereotest, and the proposed 3D stereotest results were similar with the Distance Randot stereotest. A future study including normal subjects with good stereovision should be performed, based on an elaborate design to investigate the behind/in front difference with random dot-based stereograms without contours both at near and at distance.

Our study has limitations as follows: 1) self-emitting 3D monitor may influence pupil size, retinal illuminance, and contrast sensitivity, which are the factors that can affect the level of stereopsis.[1,7,23] Although there was no significant difference in the illumination of the room between our 3D stereotest and the Randot stereotest (250 to 300 lux, both), the brightness of the self-emitting 3D monitor could have affected test results.[7] The impact of pupil size on stereoacuity may be insignificant, as there is no significant reduction in stereoacuity for pupil sizes larger than 1.5 mm.[1] The difference in retinal illuminance and contrast sensitivity caused by different monitor brightness or contrast ratios might influence the measurement of stereopsis.[7] 2) The efficacy of the new 3-D stereotest in measuring the low levels of stereopsis of ≥250 arcsec was not thoroughly evaluated, probably due to the small numbers of subjects with stereoacuity values of ≥250 arcsec. Therefore, further studies involving a larger population with a wide range of stereoacuity are needed for the validation of the new 3D stereotest.

In conclusion, we developed a new 3D display stereotest using shutter glasses that can measure a stereoacuity of 20–3000 arcsec, both at near and at distance. The display of random image patterns at every test prevented learning effects, and improved the validity of the test. Monocular cue was completely excluded by using uniformly distributed randots without a contour. Our proposed method showed good concordance with the Distance Randot stereotest compared to the conventional method. We believe that the new stereotest using 3D display could be helpful in evaluating near and distant stereoacuity in children.
